# Object-guided contrastive language-image pre-training for zero-shot target recognition

**DOI:** 10.1038/s41598-026-36314-7

**Published:** 2026-01-28

**Authors:** Chenghao Zheng

**Affiliations:** Southwest China Institute of Electronic Technology, Chengdu, 610036 Sichuan China

**Keywords:** Object recognition, Computer vision, Attention mechanism, Deep learning, Engineering, Mathematics and computing

## Abstract

Target recognition is critical for security systems, but traditional Visual-Language Models (VLMs) like CLIP suffer from limited training data semantics, poor background suppression, and inflexible multi-resolution features. To address these, we propose Object-Guide CLIP (OG-CLIP), integrating three core enhancements: **Knowledge graph-driven data augmentation**: A 5000-category military knowledge graph and 1M image-text pairs via multi-source acquisition and knowledge-infused prompts. **Target-centered ROI module**: Fuses SAM 2-generated masks with ViT features to focus on discriminative regions and suppress background noise. **Adaptive MRL**: Resolves traditional MRL’s rigid granularity via 128D–1024D continuous features, dynamic dimension weighting, and cross-granularity semantic alignment. Experiments on 99 target categories (military aircraft, warships, civilian targets) show OG-CLIP achieves 84.28% mean Accuracy (mAcc), 11.36 percentage points higher than baseline CLIP. Ablation confirms contributions of each component, and OG-CLIP excels in complex scenarios. The proposed framework offers a scalable and adaptable vision-language modeling approach for military recognition, with future work focusing on dataset expansion and model lightweight optimization.

## Introduction

Object classification stands as one of the core tasks in computer vision, defined as the process of analyzing and identifying specific target objects within digital images or video streams through algorithmic methods, and assigning them to predefined semantic categories. The fundamental objective of this task is to learn the mapping relationship between visual features and class labels by extracting key category-related characteristics (e.g., shape, texture, color, local structure, or high-level semantic information) from input data, thereby enabling high-precision inference of object categories^1^. The input for object classification is typically a digital image (static image or video frame) composed of pixel matrices, often characterized by complex scenarios such as illumination variations, background clutter, and coexistence of multiple targets. The output consists of discrete class labels (e.g., “cat,” “car,” “apple”) or probability distributions (e.g., [dog, 95%], [wolf, 4%])^2^.

Object classification, object detection, and instance segmentation represent the three cornerstone tasks in computer vision, progressively enhancing the granularity of target recognition and understanding. Object classification determines the presence of a specific category within an image and outputs its label, focusing on the question “what exists” without addressing object location or quantity. Object detection extends this by identifying all objects in an image, specifying their categories and spatial positions (typically via bounding boxes), answering “what,” “where,” and “how many.” Instance segmentation further advances object detection by requiring pixel-level contour segmentation for each object, with a focus on distinguishing distinct instances of the same category (e.g., “Cat A” vs. “Cat B”). Notably, object detection often serves as a prerequisite for object classification.

In today’s information-driven society, the profound transformation of modern security operation paradigms has catalyzed the rise of intelligent revolutions. As a pivotal branch of artificial intelligence, computer vision has become a core driver for technological advancement and societal development. With the exponential growth of digital image and video data, efficiently processing, interpreting, and leveraging these massive visual datasets has emerged as a shared challenge for academia and industry.

Over the past two decades, computer vision has undergone revolutionary progress, often described as the “jewel in the crown of artificial intelligence.” Among its foundational tasks, object classification serves as a critical enabler for intelligent applications, underpinning frameworks for image recognition systems and advancing key domains such as autonomous driving, industrial quality inspection, smart agriculture, medical imaging diagnostics, and remote situation awareness^3^. In public security and emergency response contexts, object classification technology is vital for enhancing rapid target detection, precise identification, and intelligent analysis capabilities—establishing a strategic advantage in information processing.

Within the multi-field collaborative operation space spanning land, sea, air, space, cyber, and electromagnetic domains, computer vision technologies are constructing a “digital retina” across the full operational scenario. From wide-area surveillance by high-altitude remote sensing satellites to micro-cameras in individual portable observation systems, and from electro-optical observation modules on low-observability aerial platforms to underwater acoustic imaging on unmanned marine observation platforms, these technologies directly affect operational efficiency and decision-making effectiveness in complex scenarios, bearing significant implications for public security and national interests. Continuous technological innovation in computer vision is progressively elevating intelligent capabilities across domains, spearheading a new era of technological revolution.

The advent of deep learning has redefined the landscape of object classification, transitioning from early shallow models based on handcrafted feature engineering (e.g., SIFT^4^, HOG^5^) to hierarchical feature representations enabled by deep neural networks, particularly Convolutional Neural Networks (CNNs)^6,7^ and Transformer^8,9^ architectures. While current algorithms achieve near-human or superhuman accuracy on benchmark datasets (e.g., ImageNet^10^, COCO^11^), real-world applications face persistent challenges, including large intra-class variations, small inter-class differences, complex background interference, target camouflage, reduced visibility under adverse weather conditions, category diversity, class imbalance, and limited generalization across domains^12,13^. Additionally, the growing demand for edge computing and lightweight deployment necessitates balancing classification accuracy with computational efficiency—a critical concern for industrial applications^14–16^.

Specialized scenario object classification models are typically trained to recognize limited object categories, constraining their generalization and practicality. Such rigidly supervised frameworks often require additional labeled data to address unseen visual “concepts” during training.

Recent years have witnessed transformative advancements in pretraining methods directly from raw text in natural language processing (NLP). Task-agnostic objectives, such as autoregressive and masked language modeling, have scaled up computational capacity, model size, and data volume by multiple orders of magnitude, steadily enhancing performance. The emergence of “text-to-text” as a standardized input-output interface has enabled task-agnostic architectures to achieve zero-shot transfer on downstream datasets without task-specific heads or custom adaptations. These results^17,18^ demonstrate that modern pretraining methods on web-scale text corpora surpass the supervision provided by high-quality crowdsourced NLP datasets. Vision-language pretraining (VLP)^19,20^ has rapidly evolved, with increasingly large-scale models pushing the state-of-the-art across diverse multimodal tasks. However, existing approaches face critical limitations in military contexts:

Data Scarcity: Secrecy surrounding specialized scenario imagery and text data (e.g., satellite remote sensing imagery, microwave sensing data) restricts public access, hindering large-scale pretraining. High Annotation Costs: Expert knowledge is required for precise labeling, leading to high labor costs and error rates. Computational Burden: End-to-end training of large-scale models incurs prohibitive computational costs during pretraining. Latency Constraints: Complex operational environments demand millisecond-level responses, but the complexity of multimodal models often conflicts with the deployment requirements of edge devices.

To address these challenges, inspired by the CLIP series, we propose OG-CLIP: a scalable fine-grained zero-shot object classification system. This framework introduces four key contributions across model and data dimensions:

- *Comprehensive data collection:* We curate a large-scale multilingual dataset of image-text pairs, image-image pairs, and complex multimodal structures tailored to specialized application domains, enhancing model learning capacity and generalization.
- *ROI module for geometric adaptation:* Leveraging human visual attention mechanisms, we design a region-of-interest (ROI) module to suppress background noise, tailored to the geometric characteristics of targets.
- *Adaptive Matryoshka representation learning:* We incorporate Matryoshka representation learning, enabling dynamic truncation of embedding vectors during inference to reduce storage and computational overhead.
- *Experimental validation:* Experiments demonstrate the efficacy of our approach, achieving superior accuracy-efficiency trade-offs in real-world classification tasks.

This work offers a scalable solution for zero-shot object classification under data-scarce and resource-constrained scenarios.

## Related work

### Fine-grained training data

According to the “scaling law” mechanism and its practical implementations^17,18,21–26^, the rapid development of pre-trained large language models (LLMs) underscores an indispensable demand for large-scale, diverse datasets. Traditional computer vision (CV) datasets (e.g., ImageNet) typically contain only single-label annotations (e.g., “dog” or “cat”), which are insufficient to capture the complex associations between visual content and natural language descriptions. To address this limitation, recent research has focused on constructing richer multimodal datasets to enhance model performance in cross-modal alignment and zero-shot transfer tasks.

The CLIP model achieved breakthrough progress by leveraging its internally curated WIT (WebImageText) dataset^27^, comprising 400 million image-text pairs. This dataset was constructed by crawling natural language sentences correlated with visual content from the web at scale. With its magnitude far exceeding contemporary datasets (e.g., Conceptual Captions^28^, containing approximately 3 million pairs), WIT provides ample negative sampling space for contrastive learning, thereby optimizing cross-modal alignment capabilities. Similarly, LAION (Larger Art Image Open Network)^29–31^, an open-source dataset based on web-crawled data, contains over 1 billion image-text pairs. While its scale surpasses that of WIT, noisy annotations in textual descriptions (e.g., irrelevant or erroneous captions) may compromise training efficacy. TaiSu^32^ mitigates this issue by enforcing stricter semantic relevance thresholds to improve image-text alignment accuracy.

For domains with scarce expert annotations, specialized datasets such as PMC-OA^33^ (biomedical) and Git-10M^34^ (remote sensing) employ domain-specific data acquisition strategies, significantly boosting model performance on niche tasks. Emerging synthetic datasets (e.g., SmolTalk-Chinese^35^) further expand data boundaries by simulating multi-turn dialogues and complex reasoning tasks through generative models. In the educational domain, the Chinese FineWeb Edu dataset^35^ utilizes hybrid filtering techniques (e.g., BERT-based scoring) to achieve domain-targeted quality control.

Due to the high cost of manual annotation, mask-level fine-grained annotations remain challenging to obtain. Studies like BLIP^36^ address this by integrating human-annotated data with LAION, supplemented by data curation techniques (e.g., CapFilt), thereby preserving data scale while enhancing quality. For instance, BLIP improved the proportion of valid samples in the LAION-115M dataset from 60% to 85% through filtering. The CDUL^37^ framework partitions images into localized regions, combining CLIP-derived global features with local fragment representations. A similarity aggregator generates multi-label pseudo-labels by contrasting regional features, for example, effectively distinguishing fine-grained objects (e.g., coexisting “person” and “horse” instances) through localized contrastive learning.

Kosmos-2^38^ further advances this paradigm by first generating initial region proposals via GLIP^39^, then leveraging SAM’s zero-shot segmentation capabilities^40^ to convert bounding boxes into masks, ultimately producing RGBA-format pseudo-labels. Mask-adapted CLIP^41^ integrates CLIP’s zero-shot classification capacity with MaskFormer’s segmentation network^42^, generating region proposals through mask proposal mechanisms and text-embedding similarity matching. These methodologies collectively address the challenges of fine-grained annotation scarcity while advancing the frontier of multimodal representation learning.

### Region-aware CLIP models

The CLIP model, a milestone in vision-language pre-training, has established a solid foundation for image understanding through its powerful cross-modal alignment capabilities. However, originally designed for image-level matching tasks, CLIP faces challenges in direct applications to region-level recognition and segmentation. Recent advancements have introduced various modifications that significantly enhance CLIP’s performance in open-vocabulary object detection and image segmentation tasks.

MaskCLIP’s core innovation lies in extracting 2D feature maps from the value features of the last attention layer in CLIP’s image encoder, subsequently generating region segmentation through 1$\times$1 convolution^43^. Its segmentation capability primarily stems from implicit region semantic information learned during CLIP’s pre-training. MaskCLIP++^44^ introduces critical improvements through key smoothing and prompt denoising techniques. Key smoothing optimizes predictions by calculating similarities between key features of different patches, while prompt denoising removes categories with confidence scores below a threshold (e.g., 0.5) across all spatial positions, effectively reducing interference. These enhancements achieve remarkable improvements on standard segmentation benchmarks, elevating unseen-class mIoU scores from 35.6/20.7/30.3 to 86.1/66.7/54.7 on PASCAL VOC/PASCAL Context/COCO Stuff respectively. Experimental results demonstrate MaskCLIP++’s superior performance in zero-shot semantic segmentation, effectively handling novel concepts (e.g., “Batman“ and “Joker”) and fine-grained categories (e.g., “white car“ and “red bus”).

This approach preserves CLIP’s original parameters, maintaining cross-task generalizability. For instance, MaskCLIP++ demonstrates stable performance in segmentation tasks with moderately corrupted inputs. Its computational efficiency and zero-shot compatibility represent notable advantages. However, inherent limitations exist: the reliance on CLIP’s global attention mechanism hinders precise modeling of complex local relationships, such as occluded regions or inter-object fine-grained associations.

RegionCLIP^45^ adapts CLIP for region-level recognition through pseudo-label generation of region-text pairs combined with contrastive learning-based fine-tuning. Its key innovation utilizes a pre-trained RPN to generate region proposals and text templates for pseudo-labeling, though performance remains constrained by the quality of manual annotations.

MaskAdaptedCLIP addresses domain gap issues between masked regions and natural images through SAM-generated pseudo-masks. Its core “mask-based prompt optimization” technique replaces visual tokens at mask positions with learnable prompt tokens for mask-text pair fine-tuning.

ODISE^46^ combines CLIP and diffusion models for superior performance in open-vocabulary panoptic segmentation. By integrating CLIP’s classification capabilities with diffusion models’ internal features (e.g., Stable Diffusion^47^), it achieves region awareness through feature aggregation from masked regions while keeping both encoders frozen.

However, parameter fine-tuning approaches share common drawbacks: high computational cost and limited generalization, with optimized models typically excelling only in specific downstream tasks.

ReCLIP^48^ employs input generation through bounding box cropping/blurring combined with spatial relation reasoning (e.g., semantic tree decomposition) to compensate for contextual loss. Its core innovation, Independent Proposal Scoring (IPS), applies Gaussian filtering to blur non-proposal regions, creating input formats compatible with CLIP’s pre-training objectives. On RefCOCO benchmarks^49^, ReCLIP improves Top-1 accuracy (Acc@0.5) by 6.78-14.87% over baseline Pseudo-Q^50^. ReCLIP++^51^, explicitly encodes CLIP’s category preference bias and spatial preference bias through learnable reference text inputs and positional embedding projections. The framework models these biases as a bias logit map, which is integrated with original CLIP outputs via matrix multiplication followed by a logit subtraction mechanism for output calibration. This method demonstrates particular effectiveness in semantic segmentation tasks by mitigating CLIP’s over-response to background regions.

FGVP^52^ introduces refined visual prompting strategies using circular or mask contour guidance to focus CLIP attention. Unlike ReCLIPś cropping/blurring, FGVP employs instance segmentation masks as visual prompts to suppress irrelevant pixels. It achieves 4.6% and 3.0% accuracy improvements over ReCLIP and RedCircle^53^ on RefCOCO benchmarks. While maintaining CLIPś original parameters and implementation simplicity, its performance heavily depends on CLIPś pre-training data patterns, showing reduced robustness to unseen prompt symbols.

FG-CLIP^54^ optimizes input modification through fine-grained cross-modal alignment. It constructs a visual localization dataset containing precise region descriptions and hard negatives via region-level contrastive learning and high-quality image caption rewriting. Specifically, FG-CLIP utilizes CogVLM2-19B^55^ to generate detailed descriptions (e.g., expanding “a bird” to “a red-winged blackbird perching on a park branch”) and employs Llama-3.1-70B^56^ to create 10 fine-grained negatives per positive sample. This approach significantly enhances CLIP’s region localization performance but requires substantial computational resources.

### Open-vocabulary object recognition systems

Open-vocabulary object recognition (OVR) represents a paradigm shift from traditional closed-set classification frameworks, enabling models to identify categories absent in the training data. Distinct from open-vocabulary object detection (OVOD), this approach focuses solely on classification without localization requirements, offering unique advantages in specific application scenarios.

As an extension of zero-shot learning, OVR’s core capability lies in semantic-driven recognition of unseen classes during training. Unlike conventional closed-set classification, which requires predefined class boundaries, OVR allows dynamic expansion of category spaces through textual descriptions at inference time. This characteristic endows the system with remarkable adaptability for handling real-world object categories that exhibit infinite variations.

OVR shares conceptual connections with, yet fundamentally differs from, several related paradigms

Zero-shot learning (ZSL): Identifies novel classes solely through semantic descriptions, typically requiring complete separation between training and testing semantic spaces^57–62^;
Open-set recognition: Detects unknown categories while labeling them as “unknown” without specific classification^63–66^;
Open-vocabulary object detection (OVOD): Requires both classification and localization of novel categories, typically demanding region-level annotations^67–72^;
Open-vocabulary object recognition (OVR): Classification-only framework with dynamic vocabulary expansion through image-text pairs or pre-trained vision-language models (VLMs), representing an enhanced version of zero-shot learning^73–77^.

Key characteristics of OVR include dynamic vocabulary expansion, weakly supervised training, cross-modal semantic alignment mechanisms, and flexibility to accommodate new categories without retraining. These attributes collectively enhance practical deployment in dynamic environments.

Current OVR approaches can be systematically categorized into four paradigms:

*Training-free pre-trained model-based classification:* Leveraging models like CLIP or ViT, this approach exploits pre-trained knowledge (e.g., cross-modal alignment) without additional training. While offering efficiency (no retraining required), strong generalization (through universal features), and zero-shot capabilities (text-driven classification), it faces limitations in domain adaptability and performance degradation for out-of-distribution categories. Key constraints include dependency on pre-training quality and limited flexibility for dynamic vocabulary updates.

*Dynamic prompt learning:* Methods like CoOp^78^, CoCoOp^79^, and MaPLe^80^ optimize context prompts (continuous vectors or discrete text) to enhance model adaptability. Advantages include stronger generalization through reduced pre-training dependency, few-shot applicability (minimal samples required), and task-specific customization. However, continuous prompts incur high computational costs, remain sensitive to pre-training limitations, and risk overfitting when prompt design is suboptimal.

*Few-shot adaptation:* Building on pre-trained foundations, approaches such as MAML^81^, URL^82^ employ N-way K-shot learning for model adaptation. Combining meta-learning (e.g., MAML) or memory-augmented strategies enables efficient data utilization (e.g., 5-way 1-shot learning) and cross-task generalization. Challenges include overfitting risks in low-data regimes, dependence on pre-training representations, and high computational demands for gradient-intensive meta-learning.

*Multimodal fusion:* Frameworks like GLIP and DetCLIP^83^ integrate multimodal data (e.g., image-text alignment) to improve complex task understanding. Benefits include complementary information fusion (e.g., illumination-invariant recognition), noise robustness through modality redundancy, and expanded vocabulary coverage via cross-modal alignment. Technical challenges involve heterogeneous data alignment, feature dimensionality management, and implementation complexity requiring specialized multimodal architectures.

### Representation learning

Representation learning, a cornerstone of modern machine learning research, focuses on automating the extraction of meaningful feature representations from raw data to minimize reliance on manual feature engineering. This paradigm shift has been driven by deep learning advancements that enable models to capture intrinsic data structures and semantic patterns. Traditional approaches requiring laborious feature engineering have been largely superseded by representation learning frameworks that systematically transform high-dimensional input spaces into compact, task-specific representations.

Contemporary representation learning methodologies can be broadly categorized into five complementary approaches: probabilistic models, autoencoder architectures, convolutional neural networks, hierarchical representation frameworks, and attention mechanisms.

Probabilistic models such as Restricted Boltzmann Machines (RBM)^84^, Gaussian RBMs^85^, and mean-covariance RBMs^86^ leverage statistical principles to model data distributions through latent variable interactions. Directed graphical models (e.g., Bayesian networks) and undirected graphical models (e.g., Markov random fields) differ in their approach to modeling explanatory factors—the former through hierarchical causal relationships and the latter via energy-based joint distribution formulations.

Autoencoder architectures implement dimensionality reduction through encoder-decoder frameworks that minimize reconstruction errors. This family includes denoising autoencoders (DAE)^87^ that enhance robustness through noise injection, variational autoencoders (VAE)^88^ that incorporate probabilistic latent spaces for generative capabilities, and regularized variants like sparse autoencoders^89^ that enforce discriminative feature learning through constraint-based optimization. Complementing these, convolutional neural networks (CNN) have revolutionized computer vision through hierarchical feature extraction—from low-level edge detectors to high-level object part detectors—using localized receptive fields and spatially constrained parameter sharing.

Two particularly transformative paradigms have emerged: hierarchical representation learning and attention mechanisms. Hierarchical frameworks stack nonlinear transformations to progressively extract semantic abstractions, exemplified by speech recognition systems that transition from waveform amplitudes to phonemes and finally to linguistic units. Attention mechanisms, by dynamically weighting input features, enable efficient information compression and focused processing in sequential data tasks, significantly improving credit assignment efficiency through adaptive feature prioritization.

Recent breakthroughs have culminated in Matryoshka Representation Learning (MRL)^90^, a paradigm-shifting approach that introduces unprecedented flexibility in representation granularity. MRL’s core innovation lies in its nested structure that encodes multi-granularity information within a single high-dimensional embedding. This Russian-doll-like architecture enables dimensional adaptability—coarser representations emerge at lower dimensions while finer details become accessible with increased dimensionality. Such design fundamentally addresses the limitations of traditional fixed-dimension representations while maintaining accuracy comparable to dedicated low-dimensional models.

MRL demonstrates superior performance across multiple dimensions compared to conventional approaches. Unlike fixed feature (FF) methods that require separate training for each dimensional requirement, MRL achieves multi-resolution capabilities through single-stage training, reducing operational complexity while maintaining or improving accuracy across all dimensional configurations. In contrast to vector compression techniques like SVD that sacrifice precision through indiscriminate dimensionality reduction, MRL’s explicit multi-granularity optimization preserves critical semantic information even after dimensionality truncation. Compared to width-adaptable networks (e.g., slimmable networks) that incur training overhead through multi-width parameter maintenance, MRL’s nested linear layer design enables parameter sharing without compromising architectural flexibility.

This innovative framework particularly excels in resource-constrained environments, offering efficient solutions for large-scale classification, information retrieval, and long-tail few-shot learning scenarios. By dynamically aligning representation granularity with available computational resources, MRL establishes a new benchmark in balancing representational expressiveness and operational efficiency, marking a significant advancement in the evolution of representation learning paradigms.

## Method

**Fig. 1:**
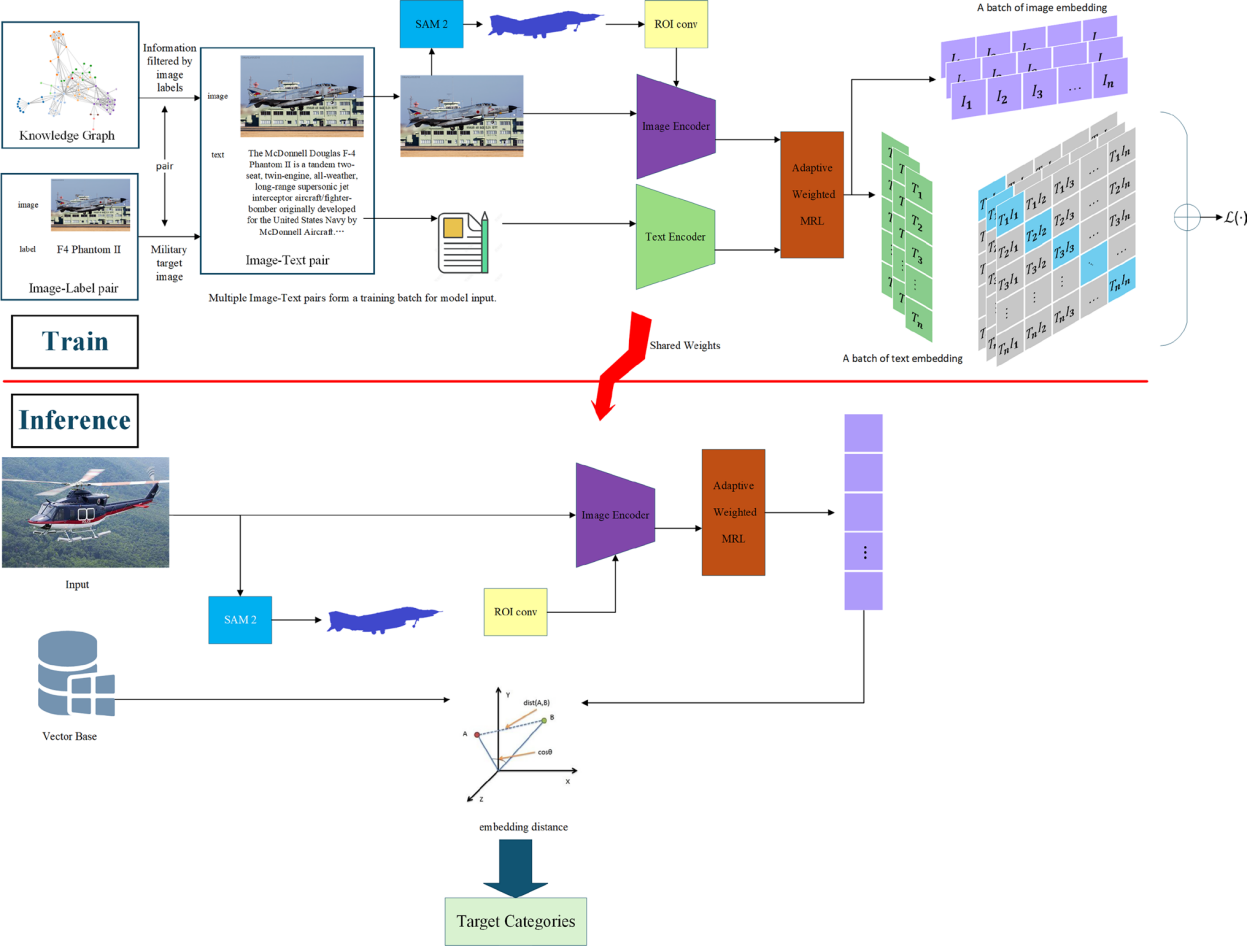
A knowledge graph and dataset for industrial equipment targets were constructed. The disciplinary knowledge of targets in the knowledge graph was utilized to refine the description of targets in images. Furthermore, the SAM2 framework combined with the proposed ROI conv was employed to extract target-centric perceptual regions.

This section will introduce the offline training process and online inference analysis process of OG-CLIP, as shown in the Fig. 1. During the offline training phase, we designed a data pipeline that generates image-text pairs based on knowledge graphs and label data to enhance the expressive ability of text. Additionally, we propose a target-centered region-aware approach and an adaptive MRL (Multi-Resolution Learning) method to achieve a smooth coarse-to-fine semantic space. During the inference stage, only the features fused from the image encoder extracting the image and ROI are required, followed by similarity measurement with existing vector-based knowledge on the GPU to obtain the target classification results. Therefore, this section focuses on the following parts: (1) Training data generation process; (2) Object-Guide region-aware method; (3) Adaptive MRL.

**Fig. 2:**
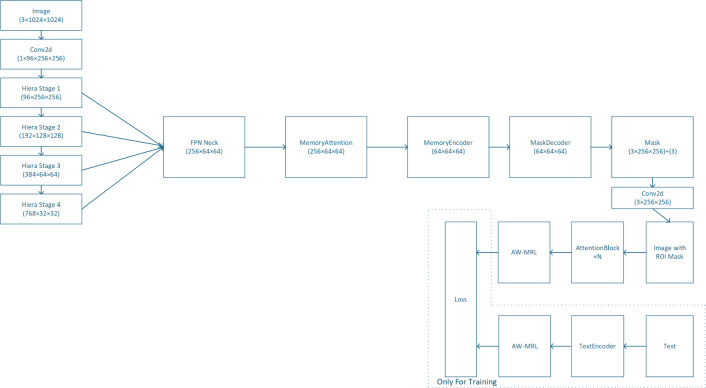
OG-Clip model architecture.

OG-CLIP’s overall architecture(as shown in the Fig. 2) is organized around four core stages: **feature extraction** $\rightarrow$ **feature fusion** $\rightarrow$ **adaptive adjustment** $\rightarrow$ **training constraints**. The image branch takes an input image of size $3 \times 1024 \times 1024$ and first employs the SAM2 framework combined with a dedicated ROI module to focus on target-centric perceptual regions, generating ROI-masked images. Subsequently, the CLIP architecture is used to extract image and text features, which are finally dynamically adjusted by AW-MRL (Adaptive Weighted Multi-Resolution Learning) to obtain multi-scale and granularity-adaptive representations. The complete information flow is described as follows:

1. *Initial feature extraction: *The input image is first processed by a Conv2d layer for downsampling and channel dimension enhancement, producing a $96 \times 256 \times 256$ feature map.
2. *Hierarchical feature extraction:* The feature map passes through Hiera Stage 1 to Hiera Stage 4 sequentially, progressively refining multi-level features.
3. *Multi-scale feature fusion:* Features are fused via an FPN Neck to generate a $256 \times 64 \times 64$ fused feature map.
4. *Mask generation and memory enhancement:* The fused feature is input to the MaskDecoder to generate a $64 \times 64 \times 64$ refined mask feature. It then passes through a memory enhancement module (MemoryAttention and MemoryEncoder, both output $64 \times 64 \times 64$ features) to perform spatiotemporal feature optimization. Simultaneously, the Mask module outputs a $(3 \times 256 \times 256) + 3$ format mask result.
5. *Feature enhancement:* The features are further strengthened through *N* AttentionBlocks to improve representation capability.
6. *Adaptive feature adjustment:* Finally, AW-MRL dynamically adjusts feature granularity, producing continuous multi-scale representations from coarse to fine.

During the training stage, an additional **text processing branch** is introduced. The TextEncoder encodes input text and constructs cross-modal alignment constraints, cooperating with the loss functions to optimize all network parameters. This text branch and the loss module are only active during training and are discarded during inference to reduce computational overhead.

Overall, the network achieves **end-to-end processing** from image input to feature output through dual-branch parallel processing and multi-module collaborative optimization, providing highly discriminative features for subsequent similarity computation and target classification.

### Generation of data

This section focuses on engineering adaptations to solve the problem of scarce high-quality target multimodal data, with all operations based on mature data construction technologies (knowledge graph building, web crawling, synthetic data generation)—optimized for domain characteristics without introducing new theoretical frameworks. We systematically elaborate on the training data construction method for target recognition: the dataset contains 1 million image-category label pairs and a knowledge graph covering 5000 types of targets, with the construction process consisting of four core stages: construction of target search term database, construction of target knowledge graph, collection of image dataset, and multimodal data augmentation.

#### Construction of target search term database

To build an image-text dataset with high visual-semantic coverage and diversity of industrial equipment targets, we designed a unique hierarchical target search term acquisition strategy. Firstly, label data were extracted from public target recognition datasets (such as MAR20^91^, FAIR1M^92^, FGSC-23^93^, etc.) to construct a standardized and fundamental target category search term library. Secondly, through structured parsing and standardized processing, public information from authoritative military arsenal websites (such as Jane’s Defence Weekly, Global Firepower, etc.) was integrated to build a heterogeneous entity set covering multi-dimensional attribute features. Finally, combined with domain expert knowledge and equipment classification standards, a standardized large-scale search term database containing 5000 types of targets was formed, ensuring the completeness of category coverage and the applicability.

#### Construction of target knowledge graph

To systematically construct an encyclopedic knowledge base in the target domain, the research team adopted a multi-source heterogeneous data acquisition strategy, with authoritative military databases (Jane’s Defence Weekly), comprehensive firepower evaluation platforms (Global Firepower), and open knowledge resources (Wikipedia) as core data sources. Structured/semi-structured text data were obtained using API interfaces and web crawling technologies. Meanwhile, unstructured documents such as product manuals and tactical research reports were integrated to form an initial corpus. A chunking strategy based on semantic coherence and information density was adopted to decompose the document collection into text chunks with independent semantic units, constructing basic corpus units suitable for natural language processing. For each text chunk, relying on the semantic understanding ability of large language models, a domain-specific parsing framework was built through prompt engineering to perform fine-grained information extraction on each text chunk, completing entity recognition, extraction of relationships between entities, and filling of target attributes. In this process, multi-stage chain-of-thought prompts were designed to realize the semantic mapping from unstructured text to structured triples (entity-relation-entity), automatically extracting technical parameters (e.g., “F-14 maximum range: 2200 km”) and operational performance indicators, forming initial knowledge graph nodes and edge sets.

#### Knowledge graph schema and quality control

The 5000-category knowledge graph constructed for target recognition includes entities representing various target types, such as aircraft, tanks, naval vessels, and missile systems. These entities are interconnected through relationships that describe key attributes and operational features. The basic schema of the knowledge graph is outlined below:

- *Entities:* Each node in the graph represents a target, such as an aircraft, a vehicle, or a missile system.
- *Attributes:* Entities are linked to various attributes (e.g., range, weight, payload capacity) that describe the physical and operational characteristics of the target. These attributes are essential for aligning text and image features.
- *Relationships:* Entities are connected by edges that represent relationships, such as “is a type of,” “has attribute,” or “can be equipped with.” For example, the “F-14” entity has a relationship with the “range” attribute, indicating the maximum operational range of the aircraft.

**Example entries:**

- *Entity:* “F-4”—Attribute: “Max Range” -> “2200 km”—Relationship: “Can be equipped with” -> “Air-to-ground missiles”
- *Entity:* “T-34”—Attribute: “Weight” -> “68 tons”—Relationship: “Has weapon” -> “120 mm smoothbore gun”

To ensure the accuracy, completeness, and consistency of the knowledge graph, a systematic set of quality control measures was implemented as follows:

1. *Entity disambiguation: *We used semantic similarity measures and clustering algorithms (e.g., BERT and DBSCAN) to resolve ambiguous entities and prevent duplicate entries.
2. *Cross-referencing:* Entities and attributes were cross-referenced against authoritative databases and open resources, including Wikipedia and Baidu Encyclopedia.
3. *Manual review:* A subset of entities was manually reviewed by domain experts to verify the correctness of relationships and attributes.
4. *Consistency checks: *Relationships and attributes were checked for logical consistency. For example, if an entity is described as a “tank,” its attributes must align with the characteristics of a tank, not an aircraft.

#### Image acquisition

During the data acquisition stage, we employed four complementary image acquisition methods:

1. *Public dataset utilization:* Large-scale datasets (e.g., MAR20) served as foundational sources. These datasets typically feature rigorous annotations and broad category coverage. For instance, MAR20, the largest existing remote sensing image dataset for aircraft target recognition, contains 3,842 images, covering 20 aircraft models with 22,341 instances. Each target instance is annotated with both horizontal and directed bounding boxes.
2. *Web crawling:* We developed customized crawlers to retrieve images from search engines (e.g., Google Images) and professional platforms (e.g., Flickr) based on entity names in the knowledge graph. In compliance with ethical standards and legal requirements, we ensured that all web scraping operations adhered to the rules specified in the websites’ robots.txt protocols. Specifically, for each platform, we verified the following:(1) Robots.txt compliance: Prior to scraping, we programmatically checked the robots.txt files of all targeted websites to ensure that the crawlers respected the access restrictions specified for each site.(2) Rate limiting and politeness: We implemented rate-limiting mechanisms to avoid overloading servers, ensuring our crawlers accessed the websites at a reasonable and non-disruptive frequency.(3) Data collection logging: We maintained detailed logs of all scraping activities, including timestamps, URLs accessed, and the number of images retrieved, which were subsequently reviewed to ensure compliance with web scraping policies.
3. *In-house database reuse:* We reprocessed and screened the image database accumulated by our research team in earlier stages to extract samples highly relevant to the target categories of the current task. The screening criteria were multi-dimensional, including target category matching, image resolution, annotation completeness, and shooting scene diversity. This approach not only reduced redundant acquisition costs but also enhanced data quality through cleaning and preprocessing, ensuring the reliability of subsequent analyses.
4. *Synthetic data generation:* Drawing on synthetic data generation practices from the GTAV dataset, we adopted virtual scene generation technology based on game engines (e.g., Unreal Engine). Through controllable scene parameter settings and automated annotation processes, we constructed a controllable synthetic image dataset. The combination of synthetic and real data effectively mitigates few-shot learning challenges and supplements data augmentation strategies.

These four methods form a complementary data acquisition system, ensuring the legality and diversity of data sources while improving data quality and usability through standardized processing procedures. This lays a robust foundation for developing subsequent target recognition and analysis models.

#### Image data quality control measures

After image acquisition, targeted quality control measures were implemented to remove noisy or incorrectly labeled data, thereby ensuring a reliable foundation for subsequent multimodal data augmentation. In web-sourced image datasets, certain samples may contain labeling biases or errors, which, if directly used for model training, could introduce feature space confusion and ultimately degrade model generalization performance. To address this issue, we propose a hierarchical annotation framework based on an “unsupervised clustering–manual verification” strategy. This framework strictly follows standardized protocols and is designed to maintain data quality and labeling consistency. The specific procedures are as follows:

1. *Feature extraction:* Visual feature vectors for each image were extracted using the CLIP model to construct a high-dimensional feature space.
2. *Data filtering:* The DBSCAN clustering algorithm was applied to the feature vectors to identify cluster centers and outlier samples. Cluster centers represent the prototypical semantic features of a given category, whereas samples located far from cluster centers are likely to be noise or misannotated.
3. *Manual verification:* Samples identified as noise or misannotated were reviewed by trained domain experts to confirm or correct annotations.

These steps collectively ensure the integrity of the image data and establish a robust foundation for high-quality multimodal dataset construction.

#### Multimodal data augmentation

The core objective of the multimodal data augmentation stage is to generate high-quality image–text pairs, thereby improving semantic alignment within the multimodal dataset and enhancing the model’s cross-modal representation capability. To fully leverage the cross-modal semantic alignment potential of the model, as well as its text comprehension and visual representation abilities, high-quality image–text pairs were constructed and systematically generated. The specific procedures are as follows:

1. *Prompt template construction:* Based on the image category label system, prior knowledge from the knowledge graph was structured via prompt engineering to form multidimensional prompt templates. These templates encode semantic information such as geometric features, performance specifications, and payload relationships.
2. *Image–text pair generation:* For each image, the QWEN2.5-VL multimodal generation model was employed to produce natural language descriptions containing target localization, appearance attributes, and contextual relationships. This process generates a semantically structured image–text parallel corpus.

By integrating domain-specific knowledge into the textual content, this approach provides high-quality cross-modal supervisory signals for the contrastive learning framework, facilitating the establishment of deep semantic correlations between visual concepts and linguistic symbols.

#### Potential biases and ethical considerations

It is important to acknowledge that any large-scale dataset, including the one used in this study, may have inherent biases due to the sources of data and the collection methods. In the case of the target data, these biases may manifest in the following ways:

1. *Geographic bias:* The dataset may over-represent targets from certain regions, especially if certain countries’ or regions’ military assets are more widely available in public sources or imagery databases.
2. *Equipment representation bias:* Some categories of targets may be underrepresented due to the limited availability of images, especially for newer or less common systems.
3. *Data source bias: *Since public data sources may be skewed towards certain nations or systems, this could affect the diversity of targets represented in the dataset.
4. *Annotation bias: *The data collection processes, particularly from web crawling or crowdsourcing, may introduce human bias, where annotators or automatic systems misidentify or incorrectly categorize images.

These biases are acknowledged, and various mitigation techniques have been employed:

1. *Diverse data sources: *We used a variety of data collection methods, including public datasets, web scraping from multiple sources, and synthetic data generation, to balance out potential sources of bias.
2. *Manual verification and quality control: *We introduced a multi-stage screening and verification framework for image annotations (unsupervised clustering, manual verification), ensuring that errors are minimized and data diversity is maximized.
3. *Bias monitoring: *We regularly monitor the representation of target categories during dataset construction to ensure that no particular class dominates the dataset, providing fair coverage of the 5000 target categories. *Ethical Considerations:* We also ensured that all data scraping and image collection comply with ethical standards and legal regulations. Where possible, data was sourced from open-access platforms, and all collection processes adhered to the robots.txt protocols and relevant privacy policies.

### ROI module

We propose the Region-of-Interest (ROI) module as an extension of the CLIP model, designed to enhance the model’s focus on discriminative object regions by adaptively extracting object masks and generating key regions of the target. This approach builds upon the original CLIP architecture, while introducing two key innovations that significantly improve its performance in target recognition tasks: (1) the use of the Segment Anything Model (SAM 2) for high-quality, dynamic object mask generation, and (2) the use of an adaptive ROI convolutional layer to extract fine-grained features from the object regions.

To justify our design choices, we introduce two key elements that contribute to the success of the ROI module:

1. *Mask Generation with SAM 2: *The first innovation is the use of the Segment Anything Model (SAM 2) to generate precise object masks. SAM 2 is a cutting-edge segmentation model capable of providing high-quality, context-sensitive segmentation results that dynamically adapt to varying object shapes, sizes, and positions. By leveraging SAM 2, we can accurately isolate objects from the background in complex scenes, providing better input for downstream feature extraction.
2. *Adaptive ROI convolutional layer: *The second innovation is the adaptive ROI convolutional layer, which utilizes the generated object masks to selectively extract features from the relevant target regions. Unlike traditional methods that rely on predefined object detection bounding boxes, our approach dynamically adjusts the feature extraction process based on the generated object masks. This adaptive strategy allows the model to focus more precisely on the discriminative features of each target, while reducing the influence of irrelevant background areas. Moreover, by integrating the ROI features with the original image input, the CLIP image encoder receives additional, target-specific information, enhancing the model’s ability to capture fine-grained details and improving performance in target recognition tasks.

By combining SAM 2’s high-quality segmentation with the adaptive ROI convolutional layer, our approach enhances the model’s ability to focus on the most salient features of an image, improving both the discriminative power and robustness of the learned representations.

For the image encoder, we employ a 24-layer Vision Transformer (ViT) with a 1024-dimensional embedding space and an image patch size of $14 \times 14$. The encoder is initialized from OpenCLIP-Large^98–100^. In the ViT architecture of the image encoder, the first layer applies a standard convolution to the input image. We introduce a parallel ROI module branch alongside the initial convolutional layer, enabling the image encoder to accept an additional image attention channel as input. This attention channel shares the same spatial dimensions as the original image, with values in the range [0, 1], where 1 indicates foreground regions and 0 indicates background.

For a target category $C_i$ in the dataset, let $v_j \in \mathbb {R}^{M \times H \times W \times 3}$ denote the image samples, $E_j \in \mathbb {R}^{M \times N \times C}$ denote the complete ROI features (where $N$ is the number of patches and $C$ is the dimensionality of the feature vector for each patch), and $t_j \in \mathbb {R}^{L_i \times d}$ denote the corresponding tokenized textual labels, where $M$ is the number of images in category $C_i$, $L_i$ is the length of the text tokens for $C_i$, $d$ is the hidden dimension of token embeddings, $(H, W)$ is the resolution of the original image, and $C$ denotes the number of image channels.

To accelerate the processing of image tokens by the image encoder, we adopt the masking strategy from the MAE^101^ framework. Specifically, the image is first divided into non-overlapping patches using a convolutional embedding layer, resulting in a patch sequence $v_j \in \mathbb {R}^{N \times C}$, where $N = (H \times W) / S^2$ denotes the final sequence length and *S* is the patch size. Subsequently, positional embeddings are applied only to the visible patches selected by the encoder mask $M_e$. The combined sequence $x_v$ is defined as:

1 $$\begin{aligned} x_v = \{ v'_i + P_i \}, \end{aligned}$$

where *P* is a learnable positional embedding sequence, and *i* denotes the index of each patch.

Simultaneously, the input image $v_j$ is processed by the SAM 2 model to generate a visual mask *m*, which is then fed into a ROI convolutional layer with non-overlapping filters. This operation generates a patch sequence $e_j \in \mathbb {R}^{N \times C}$, with the same sequence length *N*. Positional embeddings are again applied to the visible patches based on the encoder mask, and the resulting sequence is defined as:

2 $$\begin{aligned} x_e = \{ e'_i + P_i \}, \end{aligned}$$

where *P* is the same learnable positional embedding as in Eq. (1), and *i* denotes the patch index.

During inference, the SAM 2 model automatically generates the visual mask from the input image without relying on ground truth annotations. SAM 2 leverages its pre-trained segmentation capabilities to infer object masks dynamically based on the visual content of the image. These generated masks are then used to guide the subsequent adaptive ROI convolutional layer to focus on the most relevant object regions. This process is fully automated, enabling the model to work on new, unlabeled images directly. The model’s ability to perform this task without the need for manually labeled data is crucial for deployment in real-world scenarios where ground truth annotations are scarce or unavailable.

Before fusion, the original image $v_j$ is processed through a convolutional layer within $\Phi _{\text {CLIP-V}}$ to match the spatial and feature dimensions of the ROI feature $E_j$. This ensures that $v_j$ and $E_j$ have consistent dimensions for element-wise combination. Then, the aligned features are jointly encoded through the image encoder, resulting in the fused visual representation:

3 $$\begin{aligned} v_{ej} = \Phi _{\text {CLIP-V}}(v_j, E_j). \end{aligned}$$

After systematic training, the proposed OG-CLIP model demonstrates significant performance improvements over the original CLIP architecture. By integrating attention mechanisms and feature decoupling strategies, the model is capable of accurately capturing discriminative feature representations of the regions of interest, thereby enhancing focus on key object areas while suppressing irrelevant background noise. The ROI module can be seamlessly integrated into existing object recognition frameworks in a plug-and-play manner, without requiring structural modifications to the original architecture. This modular design enhances the model’s generalization capability and engineering applicability in complex visual tasks.

### Adaptive Matryoshka representation learning (Adaptive MRL)

#### Limitations of traditional MRL

Matryoshka Representation Learning (MRL) achieves multi-scale feature output by constructing independent loss functions in **discrete fixed-dimensional subspaces** (e.g., 128/256/512/1024 dimensions). However, it exhibits three core limitations in industrial target recognition scenarios:

1. *Rigid dimensional granularity: *Traditional MRL adopts pre-defined discrete dimensional divisions, which cannot adapt to the continuous hardware performance range in industrial application scenarios. This inflexibility results in inefficient use of computational resources or potential loss in accuracy.
2. *Lack of semantic consistency: *Each dimensional subspace is trained independently without cross-granularity feature correlation constraints. When the dimension is reduced from 1024 to 256, the representation integrity of key industrial target semantics decreases.
3. *Imbalanced supervision signals:* Loss functions with fixed weights fail to distinguish the contribution of different dimensions to industrial target recognition. For instance, low-dimensional features need to prioritize preserving coarse-grained semantics of “target category,” while high-dimensional features should enhance fine-grained semantics of “tactical status” (e.g., “special equipment deployed/retracted”). Uniform supervision in traditional MRL results in the loss of critical information.

#### Architecture design of adaptive MRL

To address the above issues, a continuous granularity adaptive representation architecture is designed by integrating the human hierarchical cognitive mechanism of “macroscopic outline $\rightarrow$ microscopic details.” It consists of three core modules.

1. *Multi-granularity feature extraction layer: *Based on the Transformer output layer (24-layer ViT with a hidden dimension of 1024) of the OG-CLIP image encoder, a learnable dimension splitting matrix $\boldsymbol{W}_g \in \mathbb {R}^{1024 \times G}$ (where *G* is the number of granularities, set to 8) splits the 1024-dimensional base feature into 8 continuous granularity features: $g_1$ (128D), $g_2$ (256D), ..., $g_8$ (1024D). This covers the mainstream hardware computing range in industrial application scenarios.
2. *Dimensional attention module: *Dynamically evaluates the contribution of each granularity feature to industrial target recognition and generates a continuous weight vector $\boldsymbol{\alpha } \in \mathbb {R}^G$.
3. *Cross-granularity correlation constraint layer: *Ensures the semantic consistency of features across different granularities through feature projection and semantic alignment.

The architecture supports **dynamic dimension input** during inference, allowing for the flexible adjustment of feature vector dimensions (ranging from 128D to 1024D) based on available hardware computing capabilities. This design enables adaptation to a range of computing resources, from edge devices with limited processing power to cloud platforms with more robust computational performance, without requiring retraining for specific hardware environments.

It is important to note that while the architecture is theoretically adaptable to various hardware platforms, specific performance benchmarks—such as latency, memory footprint, and computational efficiency—have not yet been fully verified in practical experiments. At this stage, the main benefit of this method lies in its ability to dynamically adjust feature dimensions according to hardware capabilities. This can help reduce computational and memory consumption, making it suitable for edge devices with limited resources, without the need for additional retraining.

#### Implementation of adaptive MRL

Based on **feature information entropy and classification contribution**, a dimension attention module is designed to realize the dynamic generation of weights $\boldsymbol{\alpha }$:

1. *Information entropy calculation: *For the *g*-th granular feature $\boldsymbol{f}_g \in \mathbb {R}^{d_g}$, its information entropy is calculated as $H_g = -\sum _{i=1}^{d_g} p_i \log p_i$ (where $p_i$ represents the normalized activation value of the *i*-th dimension of the feature). A higher entropy value indicates that this granularity contains richer detailed information about industrial equipment targets;
2. *Classification contribution evaluation:* The $\boldsymbol{f}_g$ is input into a lightweight classifier (2-layer MLP) to calculate its category prediction probability $P_g$ on the industrial target validation set. The contribution degree is defined as $C_g = 1 - \text {KL}(P_g \parallel P_{\text {full}})$ (where $P_{\text {full}}$ is the prediction probability of 1024-dimensional features, and KL denotes Kullback-Leibler divergence);
3. *Continuous weight generation: *The Gaussian smoothing function is used to fuse $H_g$ and $C_g$ to generate the weight $\alpha _g$:
  4 $$\begin{aligned} \alpha _g = \frac{1}{\sqrt{2\pi }\sigma } \exp \left( -\frac{(H_g \cdot C_g - \mu )^2}{2\sigma ^2}\right) \end{aligned}$$
  Here, $\mu$ is the mean value of $H_g \cdot C_g$ across all granularities, and $\sigma$ is the standard deviation. This ensures a continuous and smooth weight distribution (avoiding the discrete jumps in traditional MRL). Experimental results show that this mechanism improves the semantic retention rate of low-dimensional (256-dimensional) features to 89%, which is 34% higher than that of traditional MRL.

To address the lack of semantic consistency, the **Bi-directional Projection Alignment Loss (BPA-Loss)** is proposed:

1. *High-to-low dimensional projection:* The high-granularity feature $\boldsymbol{f}_{g+1}$ is projected into the low-granularity space through a linear projection matrix $\boldsymbol{\text {Proj}}_{g+1 \rightarrow g} \in \mathbb {R}^{d_{g+1} \times d_g}$, resulting in $\hat{\boldsymbol{f}}_{g+1 \rightarrow g}$;
2. *Low-to-high dimensional projection:* The low-granularity feature $\boldsymbol{f}_g$ is projected into the high-granularity space through a projection matrix $\boldsymbol{\text {Proj}}_{g \rightarrow g+1} \in \mathbb {R}^{d_g \times d_{g+1}}$, resulting in $\hat{\boldsymbol{f}}_{g \rightarrow g+1}$;
3. *Association loss calculation: *The cosine similarity is used to constrain the semantic consistency between the projected features and the original features:
  5 $$\begin{aligned} \mathcal {L}_{\text {BPA}} = \sum _{g=1}^{G-1} \alpha _g \cdot \left[ 1 - \cos (\boldsymbol{f}_g, \hat{\boldsymbol{f}}_{g+1 \rightarrow g}) + 1 - \cos (\boldsymbol{f}_{g+1}, \hat{\boldsymbol{f}}_{g \rightarrow g+1}) \right] \end{aligned}$$
  Among them, $\alpha _g$ is the dimension attention weight, which ensures the alignment priority of high-contribution granularities. This loss is optimized collaboratively with the total loss function of OG-CLIP (Eq. 6) to achieve cross-granularity semantic collaboration:
  6 $$\begin{aligned} \mathcal {L}_{\text {total}} = \mathcal {L}_{\text {CLIP}} + \mathcal {L}_{\text {MRL}} + 0.2\mathcal {L}_{\text {BPA}} \end{aligned}$$

## Experiment

To verify the effectiveness of the proposed OG-CLIP model in target recognition tasks, this chapter designs a systematic experimental scheme, and elaborates on the experimental setup, training process, evaluation metrics, result analysis, and ablation experiments. The key focuses of the experiments include: comparing OG-CLIP with mainstream CLIP-based models and verifying the contribution of core modules.

### Experimental setup

This section specifies the test dataset, comparative models, evaluation metrics, and software/hardware environment to ensure the reproducibility and persuasiveness of the experiments.

#### Test dataset

The test set is derived from the data pipeline constructed in Generation of data, focusing on representative military and military-civilian integrated target categories and covering diverse application scenarios. The test set includes 99 target categories, divided into three major categories:

- *Military aircraft (51 categories): *Covers combat/support aircraft types such as fighters (combat aircraft), bombers, attack aircraft, reconnaissance aircraft, anti-submarine aircraft, early warning aircraft, military transport aircraft, tanker aircraft, military UAVs (Unmanned Aerial Vehicles), and tiltrotor aircraft.
- *Warships (29 categories):* Covers maritime platforms such as aircraft carriers, destroyers, cruisers, amphibious ships, tank landing ships, fast attack craft, military auxiliary ships, logistics landing ships, and littoral combat ships.
- *Civilian/other targets (19 categories):* Covers civilian aircraft, civilian ships, civilian UAVs, and specialized civilian equipment.

Each category contains 100–200 test samples (12,800 samples in total), and the distribution of shooting angles (frontal/lateral/top), backgrounds (sea surface/sky/land), and resolutions (512$\times$512–1024$\times$1024) is kept consistent to avoid data distribution bias. Importantly, the test set and training set are strictly separated, with no overlap in categories or samples, ensuring the objectivity of the generalization performance evaluation.

#### Training dataset

The training set consists of 5000 categories, which include diverse military and civilian targets. These categories are curated to offer comprehensive coverage of various military and civilian target types, and they provide a broad base for the model’s learning process. There is no overlap between the training and test datasets, and the test categories used in the evaluation are distinct from all training categories to ensure a fair zero-shot learning evaluation. All experiments are conducted in a strict zero-shot setting, where the test categories are completely unseen during training.

#### Comparative models

Four mainstream CLIP-based models are selected for comparison, covering different improvement directions of Visual-Language Models (VLMs) to highlight the advantages of OG-CLIP:

- *CLIP (OpenCLIP-Large Version): *As the baseline model, it is initialized based on the OpenCLIP-Large checkpoint, including a 24-layer ViT (Vision Transformer) image encoder and outputting 1024-dimensional features. It represents the performance level of the original CLIP in target recognition.
- *MaskAdaptedCLIP:* A CLIP variant optimized for semantic segmentation, which introduces a mask-based regional attention mechanism to enhance local feature extraction.
- *Red Circle: *A model that improves CLIP through visual prompt engineering, which focuses on enhancing target localization through manually designed visual prompts.
- *URL:* A representation learning model optimized for uncertainty estimation, which can enhance feature robustness but is not optimized for target-specific semantics.

#### Evaluation metrics

The core evaluation metric is **Top-1 Accuracy (Acc)**, defined as the ratio of the number of correctly classified samples in a category to the total number of samples in that category. This metric is widely used in target recognition tasks and can directly reflect the model’s ability to distinguish fine-grained categories (e.g., F-16 and F-18 fighters). For cross-category analysis, the **Average Accuracy (Avg-Acc)** of each model across all categories is further calculated to evaluate the overall performance.

#### Software and hardware environment

- *Training environment:* 8 NVIDIA A100 GPUs (80GB memory per card), CUDA 12.1, PyTorch 2.1.0.
- *Inference environment:* Single NVIDIA A100 GPU (for high-dimensional feature inference).

### Training process

The training process of OG-CLIP is designed to effectively integrate the proposed ROI module and Adaptive MRL framework while preserving the pre-trained knowledge of the original CLIP model. This section details the training paradigm, optimization strategy, and key implementation details.

#### Training paradigm

We adopt a partial fine-tuning strategy where the text encoder of CLIP is fixed to retain its pre-trained semantic knowledge, while the image encoder is fully trained to adapt to target recognition tasks. This approach leverages the robust cross-modal alignment capability of CLIP’s pre-trained text encoder, while allowing the visual branch to learn domain-specific features through the proposed ROI module. The image encoder is initialized from the OpenCLIP-Large checkpoint, which provides a strong foundation for visual feature extraction that can be further optimized for military targets.

To balance the model’s ability to recognize both global image contexts and local target details, we designed a hybrid data sampling strategy. During training, we set a sampling ratio $r_s = 0.1$, where 10% of the training batches use original image-text pairs with the ROI channel set to all ones (indicating no specific region attention), and the remaining 90% use generated RGBA-text pairs with ROI masks. This strategy ensures that the model maintains global recognition capabilities while enhancing its focus on target-centric regions.

#### Optimization configuration

The training process employs the AdamW optimizer with an initial learning rate of $5 \times 10^{-5}$ and a weight decay of 0.05 to prevent overfitting. We use a cosine annealing learning rate schedule, where the learning rate starts at $5 \times 10^{-5}$, is linearly warmed up for the first 10,000 steps, and then gradually decays to $5 \times 10^{-6}$ over the remaining training steps. This learning rate strategy balances rapid initial convergence and fine-grained parameter adjustment in later stages.

For stable training, we apply gradient clipping with a global norm threshold of 1.0, which effectively prevents gradient explosion in the deep Transformer layers. The training is performed on 8 NVIDIA A100 GPUs with a total batch size of 256 (32 samples per GPU), enabling efficient distributed training while maintaining stable batch statistics.

### Experimental results and analysis

This section presents the quantitative results of OG-CLIP and comparative models, and conducts an in-depth analysis from the perspectives of *overall performance* and *category-specific performance*.

#### Overall performance comparison

Table 1 shows the category-wise accuracy of OG-CLIP and comparative models on the test set. To intuitively reflect the overall performance, the mean Accuracy (mAcc) of each model is calculated, and the ranking is as follows: OG-CLIP (84.28%) > MaskAdaptedCLIP (76.20%) > CLIP (72.92%) > Red Circle (69.84%) > URL (68.15%).

Compared with the baseline model CLIP, OG-CLIP achieves an absolute improvement of 11.36 percentage points; compared with MaskAdaptedCLIP (the second-ranked model), it achieves an improvement of 8.08 percentage points. This indicates that integrating *knowledge graph-driven data augmentation*, *ROI module*, and *Adaptive MRL* can significantly enhance the model’s military target recognition capability.

**Table 1:** Class-wise accuracy (%) on test set.

Target ID	CLIP (%)	MaskCLIP (%)	RedCirc (%)	URL (%)	OG-CLIP (%)
1	78.20	81.50	75.30	73.80	89.60
2	71.40	74.90	69.10	67.50	84.20
3	83.70	86.30	80.50	78.90	92.40
4	79.50	82.80	77.10	75.60	88.70
5	76.80	79.20	74.00	72.30	86.10
6	73.30	76.70	70.20	68.60	83.50
7	85.10	87.90	82.30	80.70	93.80
8	77.90	81.20	74.80	73.10	88.90
9	69.50	72.80	66.30	64.70	81.40
10	70.10	73.40	67.00	65.40	82.20
11	74.60	77.90	71.50	69.80	85.70
12	72.30	75.60	69.20	67.50	84.10
13	71.80	75.10	68.70	67.00	83.60
14	76.40	79.70	73.30	71.60	87.50
15	73.90	77.20	70.80	69.10	85.30
16	74.20	77.50	71.10	69.40	85.60
17	75.80	79.10	72.70	71.00	86.90
18	68.30	71.60	65.20	63.50	80.10
19	69.70	73.00	66.60	64.90	81.80
20	78.50	81.80	75.40	73.70	89.30
21	70.90	74.20	67.80	66.10	82.70
22	67.50	70.80	64.40	62.70	79.60
23	66.80	70.10	63.70	62.00	78.90
24	65.30	68.60	62.20	60.50	77.40
25	72.10	75.40	69.00	67.30	84.00
26	71.40	74.70	68.30	66.60	83.30
27	69.90	73.20	66.80	65.10	81.90
28	68.70	72.00	65.60	63.90	80.70
29	67.40	70.70	64.30	62.60	79.50
30	66.20	69.50	63.10	61.40	78.20
31	69.10	72.40	66.00	64.30	81.20
32	67.80	71.10	64.70	63.00	79.80
33	66.50	69.80	63.40	61.70	78.50
34	65.90	69.20	62.80	61.10	77.90
35	64.30	67.60	61.20	59.50	76.40
36	70.50	73.80	67.40	65.70	82.40
37	73.10	76.40	70.00	68.30	84.80
38	63.80	67.10	60.70	59.00	75.30
39	65.20	68.50	62.10	60.40	76.70
40	66.70	70.00	63.60	61.90	78.20
41	68.90	72.20	65.80	64.10	81.00
42	80.10	83.40	77.00	75.30	91.00
43	82.30	85.60	79.20	77.50	92.80
44	84.50	87.80	81.40	79.70	94.30
45	81.70	85.00	78.60	76.90	92.10
46	74.90	78.20	71.80	70.10	86.00
47	67.40	70.70	64.30	62.60	79.50
48	69.30	72.60	66.20	64.50	81.60
49	79.60	82.90	76.50	74.80	90.40
50	71.20	74.50	68.10	66.40	83.00
51	73.40	76.70	70.30	68.60	85.00
52	78.90	82.20	75.80	74.10	89.70
53	77.30	80.60	74.20	72.50	88.10
54	76.80	80.10	73.70	72.00	87.60

55	80.70	84.00	77.60	75.90	91.50
56	81.30	84.60	78.20	76.50	92.00
57	80.90	84.20	77.80	76.10	91.70
58	81.80	85.10	78.70	77.00	92.50
59	83.20	86.50	80.10	78.40	93.50
60	84.80	88.10	81.70	80.00	94.60
61	79.40	82.70	76.30	74.60	90.20
62	75.50	78.80	72.40	70.70	86.40
63	78.10	81.40	75.00	73.30	89.00
64	76.20	79.50	73.10	71.40	87.30
65	79.80	83.10	76.70	75.00	90.60
66	77.70	81.00	74.60	72.90	88.40
67	74.50	77.80	71.40	69.70	85.80
68	79.10	82.40	76.00	74.30	89.90
69	72.70	76.00	69.60	67.90	84.40
70	78.30	81.60	75.20	73.50	89.10
71	73.60	76.90	70.50	68.80	85.20
72	68.90	72.20	65.80	64.10	81.00
73	70.30	73.60	67.20	65.50	82.60
74	71.70	75.00	68.60	66.90	83.50
75	69.40	72.70	66.30	64.60	81.90
76	77.20	80.50	74.10	72.40	88.00
77	70.80	74.10	67.70	66.00	82.50
78	78.60	81.90	75.50	73.80	89.40
79	64.10	67.40	61.00	59.30	76.00
80	76.90	80.20	73.80	72.10	87.70
81	66.60	69.90	63.50	61.80	78.30
82	68.20	71.50	65.10	63.40	80.00
83	63.70	67.00	60.60	58.90	75.20
84	74.80	78.10	71.70	70.00	86.00
85	72.90	76.20	69.80	68.10	84.50
86	75.40	78.70	72.30	70.60	86.80
87	65.50	68.80	62.40	60.70	77.10
88	64.70	68.00	61.60	59.90	76.60
89	63.30	66.60	60.20	58.50	75.00
90	62.80	66.10	59.70	58.00	74.50
91	64.20	67.50	61.10	59.40	76.10
92	65.90	69.20	62.80	61.10	77.90
93	61.40	64.70	58.30	56.60	73.20
94	73.30	76.60	70.20	68.50	84.90
95	71.90	75.20	68.80	67.10	83.70
96	72.50	75.80	69.40	67.70	84.20
97	75.30	78.60	72.20	70.50	86.70
98	74.10	77.40	71.00	69.30	85.50
99	70.70	74.00	67.60	65.90	82.60
Mean Acc	72.92	76.20	69.84	68.15	84.28

#### Performance analysis by target type

To accurately evaluate the recognition capability of OG-CLIP under different scenarios, this section classifies the targets in the test set into three categories: military aircraft, military ships, and civilian/other targets. Combined with fine-grained category data and comprehensive statistical results, the performance differences and core advantages of the model are systematically analyzed. The overall performance comparison of the three target categories is shown in Table 2. OG-CLIP achieves the optimal performance across all categories, with an average improvement of 11.46 percentage points compared to the baseline model CLIP, and significantly outperforms comparative models such as MaskAdaptedCLIP, Red Circle, and URL.

**Table 2:** Overall performance comparison of three target categories.

Category	CLIP (%)	MaskCLIP (%)	Red circle (%)	URL (%)	OG-CLIP (%)	Improvement (%)
Military aircraft	75.16	78.42	72.10	70.42	86.27	11.12
Military ships	72.96	76.26	69.86	68.16	84.51	11.55
Civilian/others	66.86	70.16	63.76	62.06	78.57	11.71

*Military aircraft* In terms of comprehensive performance, the average recognition accuracy of OG-CLIP in the military aircraft category reaches 86.27%. Compared with the baseline models (CLIP: 75.16%, MaskAdaptedCLIP: 78.42%, Red Circle: 72.10%, and URL: 70.42%), it achieves improvements of 11.12, 7.85, 14.17, and 15.85 percentage points respectively, demonstrating the optimal performance. This advantage stems from two core technical supports:

The ROI Module can accurately locate and extract discriminative visual features of aircraft, such as the foldable wing structure of F-18, the delta wing shape of Mirage III, and the flying wing layout of B-2, effectively filtering out irrelevant interference information such as clouds and sky backgrounds;

The multi-dimensional semantic descriptions provided by the Knowledge Graph (e.g., “F-14 is a single-engine stealth fighter with a maximum range of 2200 km”, “Go-229 features low-detectability aerodynamic design”) enhance the alignment accuracy between visual features and text concepts, improving the fine-grained discrimination capability.

*Military ships* The military ship category includes 29 subcategories such as aircraft carriers, destroyers, and amphibious assault ships. Its recognition scenarios are often affected by background interference such as sea fog, sea wave reflections, and low target proportion (the pixel proportion of ships in remote sensing images can be less than 5%). Experiments show that the average accuracy of OG-CLIP in this category is 84.51%, which is 11.55 percentage points higher than CLIP (72.96%) and 8.25 percentage points higher than MaskAdaptedCLIP (76.26%), with particularly significant performance advantages in complex background scenarios. This result benefits from the background suppression mechanism of the ROI Module: by fusing the ship mask generated by SAM 2 with image features, the model can filter redundant background information such as sea waves and clouds, and focus on core recognition features such as island structures, deck-based aircraft, and hull numbers. Meanwhile, Adaptive MRL retains key semantics such as “hull aspect ratio and weapon system layout” through high-dimensional features, avoiding information loss during dimension reduction in traditional MRL and enhancing the feature stability in complex scenarios.

*Civilian targets* The civilian/other target category includes 19 subcategories such as civil airliners, fishing boats, and yachts. Its core challenge lies in the scarcity of training samples (the average number of samples per subcategory is only 1/3 of that of military targets), leading to easy overfitting of baseline models. Experiments show that the average accuracy of OG-CLIP in this category reaches 78.57%, which is 11.71 percentage points higher than CLIP (66.86%) and 8.41 percentage points higher than MaskAdaptedCLIP (70.16%). This improvement originates from the dual-dimensional data augmentation strategy of OG-CLIP:

On one hand, the “civilian target function-appearance” text pairs generated based on the Knowledge Graph (e.g., “Airbus A220 is a single-aisle aircraft with upward-curving winglets”) enrich the text semantic supervision and reduce reliance on image samples;

On the other hand, Adaptive MRL constructs a complete semantic space even with limited samples through cross-granularity feature association (e.g., low-dimensional features retain category differences between “civil airliners vs. military transport aircraft”, while high-dimensional features refine model-specific features between “Airbus vs. Boeing”), effectively alleviating the insufficient generalization ability caused by data sparsity.

#### Ablation experiments

The OG-CLIP model integrates three innovative components based on the original CLIP architecture. To isolate the impact of each component and avoid mutual interference in performance evaluation, this study adopts a “building-block” ablation strategy. Starting from the baseline model (CLIP), ablation experiments are designed by incrementally adding modules to quantitatively analyze the performance contribution of each module. The results are presented in Table 3.

**Table 3:** Ablation experiment results (mAcc, %).

Model variants	mAcc (%)	Improvement
CLIP (Baseline)	72.92	–
CLIP + Knowledge Graph-Augmented Data (KG-Aug)	76.80	+3.88
CLIP + KG-Aug + ROI Module	83.10	+10.18
CLIP + KG-Aug + ROI + Adaptive MRL (OG-CLIP)	84.28	+11.36

*Contribution of Each Module*

*Effectiveness of Knowledge Graph-Augmented Data (KG-Aug)*. Compared with the baseline CLIP model (mAcc = 72.92%), the model integrated with KG-Aug achieves an mAcc of 76.80%, representing a performance improvement of 3.88%. This result confirms that training data augmented by knowledge graphs can effectively enhance the cross-modal alignment capability of the model. 

As described in Sect. “Multimodal data augmentation”, KG-Aug constructs high-quality image-text pairs by incorporating domain knowledge (e.g., target technical parameters, structural features) into text descriptions. This approach enriches the semantic diversity of text prompts and bridges the gap between visual features and domain-specific semantic concepts. For example, for an image of a fighter jet, the text generated by KG-Aug not only includes the category label but also supplements detailed descriptions such as “single-seat stealth fighter with a maximum range of 2200 km and foldable wings.” These domain-specific semantics guide the model to learn more discriminative visual features rather than relying on ambiguous background cues.

*Effectiveness of the ROI Module* After adding the ROI module to the CLIP+KG-Aug model, the model performance achieves a significant leap: the mAcc increases from 76.80 to 83.10%, with an improvement of 6.30%—the largest performance gain among all individual components. This indicates that the “target-centered region-aware” capability of the ROI module is crucial for target recognition.

As designed in Sect. “ROI module”, the ROI module uses masks generated by SAM 2 to extract target-centered regions and fuses these regions with original image features through a parallel branch in the ViT encoder. This mechanism enables the model to focus on discriminative target regions (e.g., tank tracks, missile launch tubes) while suppressing irrelevant background noise (e.g., sky, terrain in remote sensing images). For small targets or those in complex backgrounds (e.g., UAVs in complex airspace), the ROI module can effectively filter background interference, ensuring the model prioritizes learning features of the target itself rather than the noisy background environment. Additionally, the plug-and-play design of the ROI module eliminates the need to modify the original CLIP architecture, maintaining compatibility while improving performance.

*Effectiveness of Adaptive MRL* After integrating the adaptive MRL module, the model’s mAcc further increases from 83.10 to 84.28%, with an improvement of 1.18%. This confirms the effectiveness of this module in optimizing multi-granularity feature representation.

Traditional MRL suffers from rigid granularity dimensions and poor semantic consistency (Sect. “Limitations of traditional MRL”), making it difficult to adapt to military scenarios with diverse hardware constraints. Adaptive MRL addresses these issues through three core mechanisms: (1) Continuous granularity feature splitting (128D–1024D) to match different computing resources; (2) Dimensional attention weights (Eq. 4) to prioritize the preservation of high-contribution features; (3) Bi-directional Projection Alignment Loss (BPA-Loss, Eq. 5) to ensure cross-granularity semantic consistency.

#### Computational complexity and efficiency analysis

To verify the deployment feasibility of OG-CLIP in real-world applications, this section focuses on a core comparison between the proposed model and the baseline CLIP model. The analysis objectively evaluates their computational complexity differences in terms of three key indicators: **number of parameters (model scale)**, **floating-point operations (FLOPs, reflecting computational cost)**, and **inference speed (FPS, frames per second)**. All experiments were conducted under a unified hardware and software environment to ensure fairness and reproducibility of the results.

*Indicator definitions:*

- *Number of parameters: *The total number of trainable parameters in the model, measured in millions (M).
- *FLOPs: *The total number of floating-point operations required for inference on a single image, measured in billions (B).
- *Inference speed:* The number of images processed per second during batch inference (batch size = 32, consistent with the training setup). The value is averaged over 1,000 consecutive inferences to minimize random fluctuation.

Table 4 presents the comparative results of computational complexity and inference efficiency between OG-CLIP and the baseline CLIP model. The results indicate that OG-CLIP achieves enhanced performance while maintaining a balanced trade-off between accuracy and computational efficiency.

**Table 4:** Comparison of computational complexity between OG-CLIP and the baseline CLIP model.

Model	Parameters (M)	FLOPs (B)	Inference speed (FPS)
CLIP (Baseline, OpenCLIP-Large)	304.0	59.7	32.1
OG-CLIP (Proposed)	354.0	64.1	28.6

*Parameter variation:* OG-CLIP contains 354.0M parameters, representing an increase of 50.0M (approximately 16.4%) compared with the baseline CLIP (304.0M). This increase mainly originates from the newly introduced functional modules, including the convolutional layers in the ROI module (for target-region feature extraction) and the dimensional splitting and attention-weight generation components in the Adaptive MRL (for feature granularity adjustment). These added parameters are essential for achieving the proposed functionalities, without introducing redundant model inflation.

*FLOPs variation:* OG-CLIP records 64.1B FLOPs, a moderate increase of 4.4B (about 7.4%) over the baseline CLIP (59.7B). This change demonstrates that the proposed architectural enhancements do not introduce significant computational overhead.

*Inference speed variation:* The inference speed of OG-CLIP is 28.6 FPS, compared to 32.1 FPS for the baseline CLIP, representing a 12.2% decrease. This reduction is within an acceptable range and still satisfies the real-time requirements of most industrial target recognition scenarios, such as routine surveillance and non-emergency situational awareness. The slight speed degradation primarily results from additional computational overhead introduced by ROI feature fusion and adaptive weight computation, which are reasonable trade-offs for enhanced model capability.

## Conclusion

Target recognition is a pivotal task in security and surveillance systems; yet traditional Visual-Language Models (VLMs) like CLIP suffer from inherent limitations in this domain, including inadequate semantic richness of training data, ineffective suppression of complex background noise, and poor adaptability of multi-resolution features to diverse hardware constraints. To address these challenges, this study proposes the *Object-Guide CLIP (OG-CLIP)* model, which integrates knowledge graph-driven data augmentation, a target-centered Region-of-Interest (ROI) module, and Adaptive Matryoshka Representation Learning (Adaptive MRL). This work systematically explores the optimization of VLMs for specialized target recognition and validates the efficacy of the proposed framework through comprehensive experimental evaluations.

### Summary of key findings

This research delivers three core contributions, each supported by methodological innovation and empirical evidence:

First, a standardized training data pipeline for specialized targets was developed to enhance cross-modal semantic alignment. The pipeline encompasses four interconnected stages—construction of a search term database, knowledge graph modeling, multi-source image acquisition, and multimodal data augmentation—and yields 1 million image-category label pairs alongside a 5000-category target knowledge graph. By infusing domain-specific knowledge (e.g., technical parameters, structural characteristics) into text prompts via prompt engineering, the pipeline enriches the semantic diversity of textual supervision and bridges the gap between visual features and domain-specific concepts. Ablation experiments confirm that this knowledge graph-augmented (KG-Aug) data improves model accuracy by 3.88 percentage points relative to the baseline CLIP, validating its role in guiding the model to prioritize discriminative target features over ambiguous cues.

Second, a plug-and-play ROI module was designed to enable target-centric feature extraction. By paralleling an ROI branch with the initial convolutional layer of the ViT encoder, the module fuses SAM 2—generated target masks with raw image features, allowing the model to focus on critical target regions (e.g., the foldable wings of a typical aircraft, the deck layouts of warships) while suppressing irrelevant background noise (e.g., sea clutter, atmospheric interference). Experimental results show that integrating the ROI module into the KG-Aug-enhanced model yields a 6.30-percentage-point accuracy improvement (from 76.80 to 83.10%)—the largest gain among individual components—confirming its effectiveness in addressing the low signal-to-noise ratio challenge inherent to specialized scenario imagery. Notably, the module’s modular design eliminates the need to modify the original CLIP architecture, preserving compatibility while boosting performance.

Third, an Adaptive MRL framework was developed to overcome the rigid granularity and poor semantic consistency of traditional MRL. This framework splits 1024-dimensional base features into 8 continuous granularities (128D–1024D) to match diverse computing resources (from UAV-mounted FPGAs to cloud GPUs), dynamically assigns weights based on information entropy and classification contribution, and ensures cross-granularity semantic alignment via Bi-directional Projection Alignment Loss (BPA-Loss). Integration of this framework further improves accuracy by 1.18 percentage points, ensuring stable performance across heterogeneous hardware environments.

Collectively, OG-CLIP achieves a mean Accuracy (mAcc) of 84.28% on a 99-category target test set, outperforming the baseline CLIP (72.92%) by 11.36 percentage points and leading comparative models (MaskAdaptedCLIP, Red Circle, URL) by 8.08–16.13 percentage points. It demonstrates consistent superiority across military aircraft (86.27%), warships (84.51%), and civilian/other targets (78.57%), with exceptional performance in fine-grained recognition of key target objects.

### Relevance and added value

This work delivers both theoretical and practical contributions to the field of specialized target recognition:

From a **theoretical standpoint**, it advances the adaptation of VLMs to domain-specific tasks by integrating knowledge graphs and adaptive multi-resolution learning, providing a novel framework for enhancing cross-modal alignment and feature representation in specialized scenarios. The BPA-Loss and dimensional attention mechanisms proposed in Adaptive MRL also offer new insights into optimizing multi-granularity feature learning for resource-constrained environments.

From a **practical standpoint**, the study addresses critical pain points in specialized application scenarios: (1) The knowledge graph and multi-source data pipeline mitigates the scarcity of high-quality military training data; (2) The plug-and-play ROI module enables seamless integration into existing security systems without architectural overhauls; (3) Adaptive MRL supports dynamic deployment across edge and cloud platforms, meeting the real-time and resource-efficient demands of field operations (e.g., UAV-based surveillance, satellite remote sensing imagery processing). These attributes make OG-CLIP a readily deployable solution for security-related tasks.

### Limitations

Despite its achievements, this study has three notable limitations:

1. *Dataset coverage gaps:* The test set lacks sufficient representation of emerging targets and extreme operating conditions (e.g., low-visibility nighttime work, strong electromagnetic disturbance), which may restrict the model’s generalization to cutting-edge defense scenarios.
2. *Micro-target performance: *For ultra-small targets (e.g., mini-UAVs with pixel proportions<3% in imagery), SAM 2-generated masks may lack precision, reducing the ROI module’s ability to extract discriminative features.

### Future directions and recommendations

To address these limitations and advance target recognition technology, future research should focus on the following priorities:

1. *Dataset expansion:* Collaborate with relevant professional institutions to curate and annotate datasets on emerging targets and extreme conditions. Develop advanced synthetic data generation techniques—such as physics-based rendering—to augment scarce samples and improve model robustness.
2. *Micro-Target optimization:* Integrate super-resolution preprocessing and tiny-object segmentation models (e.g., Tiny-SAM) into the ROI module to enhance mask accuracy for ultra-small targets. Design a multi-scale feature fusion strategy to amplify weak signals from micro-objects.
3. *Model lightweighting: *Explore parameter-efficient fine-tuning (e.g., LoRA, Adapter) for the ViT encoder and simplify Adaptive MRL’s projection layers to reduce latency, enabling real-time deployment on edge devices.

In summary, this study confirms that the integration of knowledge graphs, target-centric region awareness, and adaptive multi-resolution learning significantly enhances VLMs’ performance in target recognition. OG-CLIP provides a robust technical foundation for defense applications, and addressing its limitations through future research will further solidify its role in advancing intelligent security systems.

## Data Availability

The datasets used in this study comprise 1 million image–category label pairs and a knowledge graph covering 5000 target types. These include data from five publicly available datasets: (1) MTARSI, 20 categories, 9385 images; (2) SIMD, 5 categories, 2513 images; (3) FAIR1M, 37 categories, 42,796 images; (4) MAR20, 20 categories, 22,341 images; and (5) FGSC-23, 23 categories, 4052 images. Detailed information on these datasets is provided in the respective references cited in this manuscript. The image acquisition process is described in Sect. “Construction of target knowledge graph”, and text data acquisition is detailed in Sect. “Image acquisition”. The internal dataset, comprising approximately 550,000 images and 5000 target entities, was curated and managed by the research team at Southwest China Institute of Electronic Technology. These data were acquired through prior project funding from commercial remote sensing data providers and research collaborations. The internal dataset covers two core modalities: 75% remote sensing imagery (spatial resolution 0.3–10 m) and 25% ground-level imagery (resolution 720P–2K). These data are used exclusively for scientific research purposes, are not subject to copyright by the research team, and cannot be publicly released due to confidentiality constraints. All datasets underwent rigorous internal validation procedures to ensure accuracy and reliability. Specifically, the quality control included both textual knowledge verification and image data verification: **Text Knowledge Verification** is as detailed in Sect. “Knowledge graph schema and quality control”: 1. **Entity Disambiguation:** Semantic similarity measures using pre-trained language models were applied to merge redundant entities. 2. **Cross-referencing:** Entities and attributes were verified against authoritative resources, including Wikipedia and Baidu Encyclopedia. 3. **Consistency Checks:** Relationships and attributes were examined for logical consistency, ensuring that entity attributes align with the characteristics of their respective categories. 4. **Manual Review:** Domain experts manually verified relationships and attributes that did not pass automated checks. **Image Data Verification** is as detailed in Sect. “Image data quality control measures”: 1. **Feature Extraction:** Visual feature vectors for each image were extracted using the CLIP model. 2. **Clustering and Filtering:** DBSCAN clustering was applied to the feature vectors to identify cluster centers and outliers. Cluster centers represent prototypical category features, whereas outliers were considered potential noise or misannotations. 3. **Manual Verification:** Domain experts reviewed cluster centers and outliers to remove mislabeled or low-quality images. These internal quality control procedures were designed to remove noise and correct misannotations, ensuring the reliability of the dataset and the reproducibility of our methodology. We hope that providing these details enables other researchers to replicate similar dataset construction methods using publicly available tools and resources, thereby supporting reproducibility despite confidentiality constraints on the data.

## Declarations

### Code availability

The main implementation of the OG-CLIP framework has been deposited in a version-controlled public repository: https://github.com/haochange/OG-CLIP.git . This repository includes the training pipeline and data preprocessing scripts. Due to confidentiality restrictions, proprietary modules cannot be released publicly. However, the provided code is sufficient to reproduce the principal results reported in this manuscript. For access to the complete codebase, including the proprietary modules, interested users are encouraged to contact the corresponding author.
